# Flexible nets: disorder and induced fit in the associations of p53 and 14-3-3 with their partners

**DOI:** 10.1186/1471-2164-9-S1-S1

**Published:** 2008-03-20

**Authors:** Christopher J Oldfield, Jingwei Meng, Jack Y Yang, Mary Qu Yang, Vladimir N Uversky, A Keith Dunker

**Affiliations:** 1 Center for Computational Biology and Bioinformatics, Indiana University Schools of Medicine and Informatics, 410 W. 10^th^ Street, Indianapolis, IN 46202, USA; 2 Institute for Biological Instrumentation, Russian Academy of Sciences, 142290 Pushchino, Moscow Region, Russia

## Abstract

**Background:**

Proteins are involved in many interactions with other proteins leading to networks that regulate and control a wide variety of physiological processes. Some of these proteins, called hub proteins or hubs, bind to many different protein partners. Protein intrinsic disorder, via diversity arising from structural plasticity or flexibility, provide a means for hubs to associate with many partners (Dunker AK, Cortese MS, Romero P, Iakoucheva LM, Uversky VN: Flexible Nets: The roles of intrinsic disorder in protein interaction networks. *FEBS J *2005, 272:5129-5148).

**Results:**

Here we present a detailed examination of two divergent examples: 1) p53, which uses different disordered regions to bind to different partners and which also has several individual disordered regions that each bind to multiple partners, and 2) 14-3-3, which is a structured protein that associates with many different intrinsically disordered partners. For both examples, three-dimensional structures of multiple complexes reveal that the flexibility and plasticity of intrinsically disordered protein regions as well as induced-fit changes in the structured regions are both important for binding diversity.

**Conclusions:**

These data support the conjecture that hub proteins often utilize intrinsic disorder to bind to multiple partners and provide detailed information about induced fit in structured regions.

## Background

Protein-protein interaction (PPI) networks integrate various biological signals including those used for energy generation, cell division and growth to give a few notable examples. The architectures of the PPI networks indicate that they are nearly scale free [1-8]. That is, a log-log plot of the number of nodes versus the number of links (or interactions) at each node gives a straight line with a negative slope. The negative slope means that these sets of interactions contain a few proteins (hubs) with many links and many proteins (non-hubs) with only a few links. The term ‘hub protein’ is relative to the other proteins in a given PPI network, with no agreed upon number of links separating hubs and non-hubs.

Several networks such as the internet, cellular phone systems, social interactions, author citations, and so on, exhibit scale-free architecture. With regard to PPIs, scale-free network architecture is suggested to provide several biological advantages. For example, given the small fraction of hub proteins, random deleterious mutations will more likely occur in non-hub proteins. The elimination of the functions of such non-hub proteins typically have small effects and so, generally, are not serious. In contrast, a deleterious mutation of a hub protein is more likely to be lethal [4-9]. Another advantage is that signals can traverse these networks in a small number of steps, so signal transduction efficiency is improved compared to that expected for random networks [7].

Understanding PPI network evolution across different species is an important problem [10-13]. From this body of work, hub proteins appear to evolve more slowly than non-hub proteins, an observation that is consistent with Fisher's classic proposal that pleiotropy constrains evolution [14,15]. Some proteins have multiple, simultaneous interactions (“party hubs”) [16] while others have multiple, sequential interactions (“date hubs”) [16]. Date hubs appear to connect biological modules to each other [17] while party hubs evidently form scaffolds that assemble functional modules [16].

The idea that PPI networks use scale-free network topology is receiving considerable attention, but some caution is in order. Currently constructed networks are noisy, with both false positive and false negative interactions [8,18-20]. Also, network coverage to date [21,21-24] is not sufficient to prove scale-free architecture [25]. Whether PPI networks are truly scale-free or only approximately so, it nevertheless appears to be true that a relatively small number of proteins interact with many partners, either as date hubs or party hubs, while many proteins interact with just a few partners.

The ability of a protein to bind to multiple partners was suggested to involve new principles [26]. Indeed, neither the lock-and-key [27] nor the original induced-fit [28] readily explains how one protein can bind to multiple partners. Note that the original induced fit mechanism was defined as changes in a structured binding site upon binding to the partner [28], changes that are analogous to a glove altering its shape to fit a hand. On the other hand, both theoretical and experimental studies over many years suggested that natively unstructured or intrinsically disordered proteins form multi-structure ensembles that present different structures for binding to different partners [29-35]. Based on these prior studies, we proposed that molecular recognition via disorder-to-order transitions provides a mechanism for hub proteins to specifically recognize multiple partners [36]. We pointed out earlier that intrinsic disorder could enable one protein to associate with multiple partners (one-to-many signaling) and could also enable multiple partners to associate with one protein (many-to-one signaling) [35].

Recent bioinformatics studies support the importance of protein disorder for hubs [37-41]. While disorder appears to be more clearly associated with date hubs [39,41] than with party hubs, some protein complexes clearly use long regions of disorder as a scaffold for assembling an interacting group of proteins [42,42-50]. Thus, the importance of disorder for party hubs needs to be examined further. Additional evidence for the importance of disorder for highly connected hub proteins comes from a structure-based study of the yeast protein interaction network [51]. The authors considered only interactions that could be mediated by domains with known structures and found that the degree distribution of the resulting network contained no proteins with more than 14 interactions, which is more than an order of magnitude less than is observed in one unfiltered, high confidence dataset (Jake Chen, personal communication). This result indicates that a structure-based view of hub proteins is insufficient to explain the multitude of partners that interact with hub proteins.

To improve understanding of the use of disorder for binding diversity, we studied two prototypical examples: p53 and 14-3-3. Both are hubs that are clearly involved in crucial biological functions. For example, p53 is a key player in a large signaling network involving the expression of genes carrying out such processes as cell cycle progression, apoptosis induction, DNA repair, response to cellular stress, etc. [52]. Loss of p53 function, either directly through mutation or indirectly through several other mechanisms, is often accompanied by cancerous transformation [53]. Cancers with mutations in p53 occur in colon, lung, esophagus, breast, liver, brain, reticuloendothelial tissues and hemopoietic tissues [53]. The p53 protein induces or inhibits over 150 genes, including *p21*, *GADD45*, *MDM2*, *IGFBP3*, and *BAX*[54].

The four regions or (not necessarily structured) domains in p53 are the N-terminal transcription activation domain, the central DNA binding domain, the C-terminal tetramerization domain, and the C-terminal regulatory domain. The last two could be considered to be a single C-terminal domain with two subregions. The transactivation region interacts with TFIID, TFIIH, Mdm2, RPA, CBP/p300 and CSN5/Jab1 among many other proteins [52]. The C-terminal domain interacts with GSK3β, PARP-1, TAF1, TRRAP, hGcn5, TAF, 14-3-3, S100B(ββ) and many other proteins [55].

As for 14-3-3 proteins, they contribute to a wide range of crucial regulatory processes including signal transduction, apoptosis, cell cycle progression, DNA replication, and cell malignant transformation [56]. These activities involve 14-3-3 interactions with various proteins in a phosphorylation-dependent manner. More than 200 proteins have been shown to interact with members of 14-3-3 family [57-59], with these 14-3-3-interacting proteins amounting to approximately 0.6% of the human proteome [59]. One proposed functional model is that 14-3-3 binds to the specific target as a molecular anvil causing conformational changes in the partner. In their turn, these changes can affect enzymatic (biological) activity of a target protein, or mask or reveal specific motifs that regulate its localization, activity, phosphorylation state, and/or stability [60].

The 14-3-3 protein has at least nine sequence isomers, called α, β, γ, δ, ε, η, σ, τ, and ζ [61]. All isomers are structured dimers with grooves that bind to more than 200 different partners, and the different partners have different sequences for their binding regions. Screening experiments have identified individual peptides that bind to all the different isomers, suggesting that the binding grooves in the different isomers have some common features [62]. A recent bioinformatics study suggests that the partners of 14-3-3 utilize intrinsic disorder for binding [63].

The interactions of p53 and 14-3-3 with their partners as reported previously [61,64-79] are examined herein but from an order-disorder point of view. In the case of p53, different regions in the disordered tails enable this protein to bind to multiple partners at the same time. In addition, one single region of disorder adopts clearly different secondary structures and uses the same amino acids to different extents in different binding interactions. For this case the plasticity of the disordered region clearly enables the binding to multiple partners. In the case of 14-3-3, the different partners have distinct sequences. Their interactions with 14-3-3 show characteristics, such as hydrogen bonds between side chains of 14-3-3 and the backbone of the partners and such as hydrogen bonds between the backbone of the partners and water, indicating that the two partners were very likely unfolded in water just prior to association with 14-3-3. The distinct sequences of the partners do not adopt identical backbone structures, and the various side chain interactions between 14-3-3 and the two different partners involve induced-fit adjustments of the 14-3-3 structure. Overall, these studies show how the plasticity of disordered proteins is used to enable the binding diversity of hub proteins, both for a single disordered region binding to multiple partners and for multiple disordered regions binding to the same partner. An earlier, less complete version of this work was reported at the Biocomp’07 meeting [80].

## Results

### Intrinsic disorder and the molecular interactions of p53

**Figure 1:**
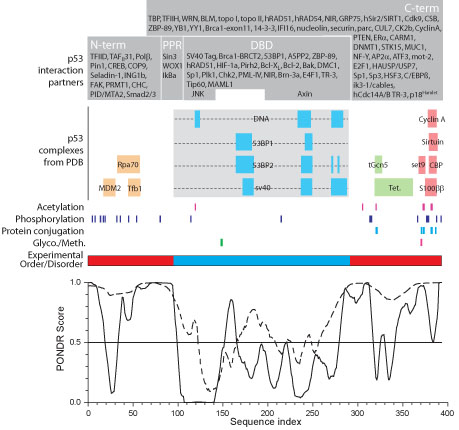
**Summary of p53 interactions and structure**. Dark gray boxes indicate the approximate binding regions of p53's known binding partners. The regions of p53 represented in structure complexes in PDB are represented by horizontal bars, labeled with the name of the binding partner. For the DBD, the extent of the globular domain is indicated by the light grey box, where the internal horizontal bars indicate regions involved in binding to a particular partner. Post translational modifications sites are represented by vertical ticks. Experimentally characterized regions of disorder (red) and order (blue) are indicated by the horizontal bar. Finally, predictions of disorder (scores > 0.5) and order (scores < 0.5) are shown for two PONDR predictors: VLXT (solid line) and VSL2P (dashed line). All, features are presented to scale, as indicated by the horizontal axis. The p53 interaction partners and post translational modification sites have been adapted from Anderson & Appella [55].

The p53 molecule interacts with many other proteins in order to carry out its signal transduction function. A number of these are downstream targets, such as transcription factors, and others are activators or inhibitors of p53's transactivation function. Many of these interactions have been mapped to regions of the p53 sequence (Figure 1, gray boxes): the N-terminal domain (i.e., the transactivation domain), the C-terminal domain (i.e., the regulatory domain), and the DNA binding domain (DBD). These domains have also been characterized in terms of their structure or lack thereof (Figure 1, red (disordered) and blue (structured) segments), where the DNA binding domain is intrinsically structured and the terminal domains are intrinsically disordered [81,82]. While the tetramerization domain is structured, the structure is acquired upon the formation of the complex. Additionally, multiple different posttranslational modifications have been identified in p53 (Figure 1, vertical ticks). These modifications are relevant here because they are a common method for altering protein interactions.

Comparing the regions of order and disorder reveals a strong bias towards the localization of the interactions within the intrinsically disordered regions. Overall, 60/84 = 71% of the interactions are mediated by intrinsically disordered regions in p53. A bias toward intrinsically disordered regions is even more pronounced in the sites of posttranslational modifications, with 86%, 90%, and 100% of observed acetylation, phosphorylation, and protein conjugation sites, respectfully, found in the disordered regions. This is consistent with previous observation of a strong bias for post translational modifications toward intrinsically disordered regions [83]. This concentration of functional elements within intrinsically disordered regions compares to just 29% of the residues being disordered [36]. Clearly, p53 exhibits a highly biased use of disordered regions for mediating and modulating interactions with other proteins.

In addition to experimentally characterized disorder, predictions of intrinsic disorder for p53 using both PONDR VL-XT [84] and VSL2 predictions [85] were carried out (Figure 1, graph). The latter is one of the highest accuracy prediction algorithms available [86], whereas the former has been observed to be especially useful in identifying binding regions within longer regions of disorder [87-89] and to be much better at identifying such sites as compared to a number of different disorder predictors [90]. Both predictors give good agreement with the experimental determination of intrinsic disorder [83,91-109], and in the case of p53 both of their predictions agree well with experimental characterization.

### Analysis of associations involving p53 using 3D structures

The structures of 14 complexes between various regions of p53 and unique binding partners have been determined (Figure 1, horizontal bars). For 10 of these partners, the interactions are mediated by regions experimentally characterized as intrinsically disordered, where PONDR VL-XT detects the majority of these binding regions as short predictions of order within a longer prediction of disorder. These structures are complexes between p53 and endogenous partners: cyclin A [64], sirtuin [65], CBP [66], S100ββ [67], set9 [68], tGcn5 [69], Rpa70 [70], MDM2 [71], Tfb1 [72], and itself [73]. The remaining 4 interactions are mediated by the structured DBD, namely between p53 and 3 endogenous partners – DNA [74], 53BP1 [75], and 53BP2 [76] – and one exogenous partner – the large-T antigen (LTag) from simian virus 40 [79].

Protein complexes can be formed from the association of structured proteins, by the folding of one disordered protein onto the surface of a structured partner, or by the coupled folding and binding of intrinsically disordered proteins [110-117]. Nussinov and collaborators [117] showed that a plot of normalized monomer area (NMA) versus normalized interface area (NIA) nicely separates complexes formed from structured proteins as compared to complexes formed from unfolded proteins by coupled binding and folding. That is, associations of structured proteins exhibit small NMAs and NIAs and so lie near the origin of the NMA-NIA plot. Conversely, complexes formed by coupled binding and folding have much larger NMAs and NIAs, and so are spread out and lie far from the origin of the NMA versus NIA plot. Indeed, a linear boundary separates the two groups [117]. IT should be emphasized that the NMA-NIA plot approach is a global measure of a proteins order-disorder monomeric state, and has not been characterized on local order-disorder transitions (e.g. disordered binding loops in an otherwise well ordered protein).

**Figure 2:**
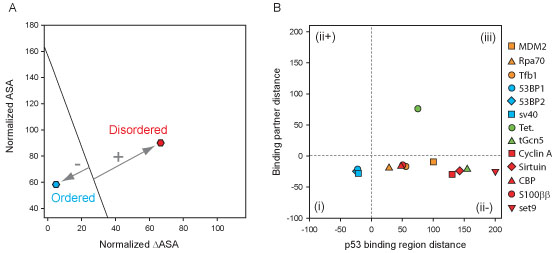
**Double NMA-NIA plot for p53 complexes.** (A) The definition of boundary distance used in the double NMA-NIA plot, where ordered structures have a negative boundary distance and disordered structures have a positive boundary distance. (B) The double NMA-NIA plot for the p53 structures shown in Figure 1, with the exception of DNA-bound p53.

As described in more detail in the implementation, by developing two separate NMA-NIA plots, one for each partner of a complex (Figure 2A), and then by determining the distance to the linear boundary in each plot, a double NMA-NIA plot (Figure 2B) can be produced. Interacting pairs can be divided into the 3 groups given above, namely: (1) both partners are structured (region (i) of 2B), i.e. both distances are negative; (2) one partner is structured and the second partner is disordered, i.e. the ordered partner has a negative distance and the disordered partner has a positive distance (regions (ii+ and ii-) of 2B); and (3) both partners are intrinsically disordered, i.e. both distances are positive (region (iii) of 2B).

A double NMA-NIA plot was calculated for 13 of the p53 complex structures (Figure 2B). The p53-DNA complex was excluded since the NMA-NIA analysis is not relevant for nucleic acids. In the general case, the distinction between the distances of the two partners is arbitrary, so that the double NMA-NIA plot is symmetric about the diagonal. However, here we restrict the p53 distance to one axis, so that group (2) is split into two sub groups (regions (ii+ and ii-)): the p53 segment is disordered and the partner is ordered (region (ii-)) and the p53 region is ordered and the partner is disordered (region (ii+)). One interaction, the formation of the p53 tetramer, is in the third group (region (iii)) and so therefore likely involves an association between two disordered partners. This is consistent with experimental data [81]. At the opposite side of the spectrum, the three protein-protein complexes involving the p53 DBD domain are in group 1 (region (iii)), indicating that all three are ordered prior to binding, which is consistent with the solution of structures for identical or homologous monomeric domains (e.g. p53 DBD [118], 53BP1 BRCT domain [119], 53BP2 SH3 domain [120], and LTag [79]). The other nine p53 complexes found so far in the PDB are all in the group 2 quadrant (that is, in region (ii-), and so all likely involve a disordered region of p53 associating with a structured partner. These results are likewise consistent with experimental data. That is, these p53 regions are disordered in the unbound state [81,82], and the isolated partners appear to be structured: MDM2 [121], Rpa70 [70], Tfb1 [72], tGCN5 [122], Cyclin A/CDK2 [123], sirtuin [124], CBP [125], S100ββ [126] and set9 [127]).

In summary, these data point out the importance of disorder-to-order transitions for many of the structurally characterized interactions involving the p53 hub protein. While many previous studies discuss these same interactions, to our knowledge the importance of disorder has not been emphasized in those previous studies.

### Analysis of multiple specificities in the p53 C-terminus

**Figure 3:**
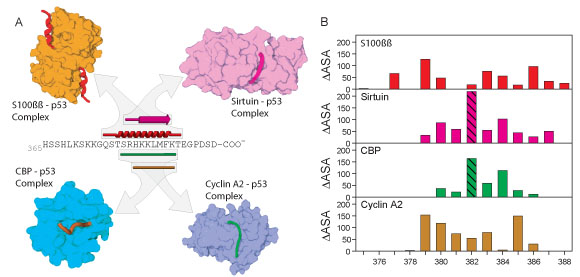
**Sequence and structure comparison for the four overlapping complexes in the C-terminus of p53**. (A) Primary, secondary, and quaternary structure of p53 complexes. (B) The ΔASA for rigid association between the components of complexes for each residue in the relevant sequence region of p53. The two hatched bars indicate acetylated lysine residues.

So far, complexes involving one region of the p53 sequence bound to four different partners have been determined and deposited in the PDB. This region is from residue 374 to 388 in the p53 sequence bound to one of the following: cyclin A [64], sirtuin [65], CBP [66], or S100ββ [67]. The regions that mediate these interactions and their respective secondary structures were mapped precisely to the p53 sequence (Figure 3A). Although slightly different residues of the p53 sequence are used in each interaction, there is a very high degree of overlap, with a span of 7 core residues being the same (Figure 3A). Interestingly, the four complexes display all three major secondary structure types. The core span becomes a helix when binding to S100ββ, a sheet when binding to sirtuin, and a coil with two distinct backbone trajectories when binding to CBP and cyclin A2 (Figure 3A).

Because the secondary structures are distinct, it seems likely that p53 utilizes different residues for the interactions with these four different partners. To examine this, the buried surface area for each residue in each interaction was quantified by calculating the ΔASA (Figure 3B). Different amino acid interaction profiles are seen for each of the interactions, showing that the same residues are used to different extents in the four interfaces. The particularly large ΔASA peaks for K382 in complexes with CBP and sirtiun (indicated by the hatched bar) are due to extra buried areas arising from the acetylation of this residue. This highlights the importance of posttranslational modification for altering PPI networks.

### Analysis of multiple specificities of the p53 DBD

**Figure 4:**
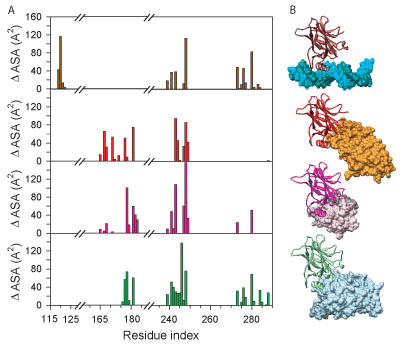
**p53 DBD interaction with different binding partners**. The interaction profiles (A) and rendered structures (B) for the four unique complexes of the p53 DBD. Rendered structures depict p53 as a ribbon and each interaction partner as a molecular surface. The interaction profile-structure pairs are (from top to bottom): p53-DNA, p53-53BP1, p53-53BP2, and p53-sv40.

The p53 molecule contains another set of overlapping interactions that contrasts with those at the C-terminus. These interactions are mediated by the DNA binding domain and include interactions with DNA [74], the BRCT domain of 53BP1 [75], the SH3 domain of 53BP2 [76], and the large T-antigen (LTag) of Simian Virus 40 [79]. Here we compare these four interactions using the methods described in Figure 4.

The structures of the p53-DNA, the p53-53BP1, the p53-53BP2, and the p53-LTag complexes are shown (Figure 4B). While all of the ligands are different, they all bind to basically the same region of p53.

Comparison of the interface profiles of the four complexes (Figure 4A) shows a large difference in the pattern of interface residues used by p53. For instance, there are several residues at the N-terminal end of the DBD which are only found in interaction with DNA. Similarly, interface residues near the C-terminal end participate in binding to different extents in three interactions, but not at all in the p53-53BP1 interaction. The differing usage of residues in each interaction is the most prevalent feature of this data. However, there are also several residues contributing an exceptionally large amount of surface area in each complex (*e.g.*, M243 and R248).

**Figure 5:**
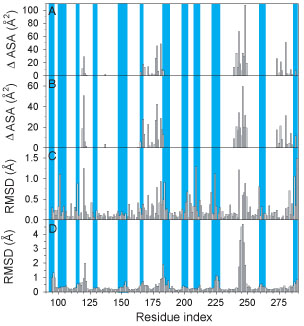
**Comparison of residue interactions with structural differences for bound p53 DBD**. The average (A) and standard deviations (B) were calculated over the four interaction profiles of the p53 DBD shown in Figure 4. These are shown aligned with the side chain RMSF (C) and the backbone RMSF (D) calculated from the four structures of bound p53 DBD. Regions of residues that are highly exposed to solvent in all complex structures are indicated by the blue-shaded regions.

While the focus of this paper is on the roles of disorder in the interactions involving two different hub proteins, the DNA binding domain of p53 presents the opportunity to study structural changes involving one structured region binding to several different structured partners. For this purpose, we compiled a 4-panel set of plots for characterizing the induced fit as one protein binds to different partners (Figure 5). These panels show the average interface area (Figure 5A), the standard deviation of the interface area (Figure 5B), the differences in side chain conformation (Figure 5C), and differences in backbone conformation (Figure 5D). Furthermore, regions that are highly exposed to solvent are also indicated (Figure 5, blue shading), so that structural differences due to interactions can be distinguished from those due from intrinsic flexibility – disordered loops – or crystallization artifacts.

This induced-fit profiles exhibit a number of interesting features (Figure 5). The most striking of these is the region from residue 240 to residue 250. This region shows a large and variable interaction interface, which is associated with large side chain and backbone conformational differences. This is true also of a smaller region around residue 120. Other interaction regions show only side chain conformational differences associated with variable interface areas. Other conformational differences observed are limited regions of high solvent exposure, which suggests that these changes are due the details of the crystallization conditions more than interaction with a particular binding partner.

Together, these results suggest that multiple partners of p53 are accommodated by reusing similar binding interfaces. This is facilitated by small scale or large scale structural differences, which range from differences in side chain conformation to backbone rearrangements. It should be noted that this differs from our finding in a more limited analysis on only the p53-53BP1 and -53BP complexes [80].

### Analysis of the multiple specificities of 14-3-3

Five different 3-D structures of the 14-3-3ζ protein bound to distinct partners were found in PDB. These partners include a peptide from the tail of histone H3 [128], serotonin N-acetyltransferase (AANAT) [77], a phage display-derived peptide (R18) [78], and motif 1 and 2 peptides (m1 and m2, respectively) [61]. For AANAT, only the region within the canonical 14-3-3 binding site is included in our analysis with the globular region being deleted. Two additional structures were not included because they were either unsuitable for structural analysis or were highly redundant with another structure. All peptides are phosphorylated in their respective structures except R18, which contains a glutamate in place of the phosphoserine.

**Figure 6:**
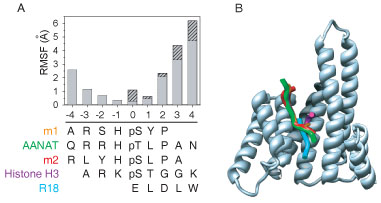
**Sequence and structure for five peptides bound to 14-3-3ζ.** (A) Sequence alignment of the bound peptides and the RMSF of their conformations. Solid grey bars give the RMSF for four peptides – excluding R18 – and the hatched bars give the RMSF for all five peptides. (B) Aligned ribbon representations of the structures of the five peptides, which were aligned through multiple alignment of their respectively bound 14-3-3 domains, show along with a representative ribbon representation of a 14-3-3 domain.

The five bound peptides sequences were aligned structurally as described in the methods. Likewise, the 14-3-3 domain structures were independently aligned, without considering the bound peptides. Next the 14-3-3 alignment was anchored manually by the observed correspondence of the bound peptide C_α_ atoms at the 0 and -1 positions and by extending the alignment without gaps from the anchor positions, thereby giving the final structural alignment (Figure 6A). In terms of sequence, the R18 sequence has no identical positions to any other peptide. The number of identities between the other peptides range from 1 to 4.

The high overlap in the backbone trajectories of the 5 peptides from position -3 to 1 but large divergences at either end of the structural alignment (Figure 6A). This divergence at the ends is apparent qualitatively in the superimposed structures of the five peptides (Figure 6B). Structural divergence and sequence variability are loosely correlated, where positions with 3 identical residues have a lower divergence than those with no identical residues. This suggests that 14-3-3 may use different binding pocket residues to interact with different peptide residues. The R18 sequence, which is divergent from the others, makes a large contribution to the estimated RMSF values (indicated by the cross-hatched bars, Figure 6A).

**Figure 7:**
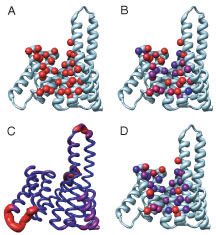
**Peptide binding residues of 14-3-3ζ.** (A) The C_β_ atoms of all residues involved in binding in any of the five peptide bound structures are shown (red) along with the rest of the backbone (light blue ribbon). (B) The standard deviation in the area bound on complex formation is displayed by coloring the C_β_ atoms of peptide binding residues on a gradient, from a standard deviation of 0Å^2^ (blue) to 10Å^2^ and greater (red). (C) The backbone RMSF of the 14-3-3 domain calculated over C_α_ atoms displayed as a color and radius gradient, from an RMSF of 0Å (blue, 0.25Å) to an RMSF of 2.0Å and greater (red, 2.0Å). (D) The side chain RMSF is displayed by coloring the C_β_ atoms of peptide binding residues on a gradient, from a RMSF of 0Å (blue) to an RMSF of 0.50Å and greater (red). All parameters were calculated using all five of the peptide-14-3-3 complexes.

The factors contributing to the ability of 14-3-3 to bind to distinct peptides were estimated by a detailed structural analysis. The peptide binding residues of 14-3-3 are located primarily in a central cleft, made up of four helices (Figure 7A), which has been noted previously by several researchers. The standard deviation of ΔASA for the peptide binding residues (Figure 7B) show that the residues with the most binding variability are located at either end of the central cleft, which is consistent with the variation of peptide backbone trajectories in these regions. Backbone variability in bound 14-3-3 structures (Figure 7C) is restricted to the ends of most of the binding cleft helices. These observations suggest that large a conformational change in 14-3-3 is not necessary for multiple specificities, although some small adjustments at the ends of binding helices may be necessary.

To assess the role of side chain conformational changes in peptide binding, the RMSF of side chain atoms was calculated (Figure 7D). The side chain RMSF and standard deviation of ΔASAs give similar indications for many binding site residues, where residues used inconsistently across multiple complexes are the most likely to undergo conformational rearrangement. These are the same residues that are located at the broadest parts of the binding site. However, a few residues deep in the binding grove show both consistent participation in the binding interface and variable side chain conformation. These observations suggests that the primary, high level mechanisms of 14-3-3 multiple specificity are a broad binding site that allows multiple trajectories (and therefore interaction with different residues) and side chain rearrangement to accommodate different peptide sequences.

**Figure 8:**
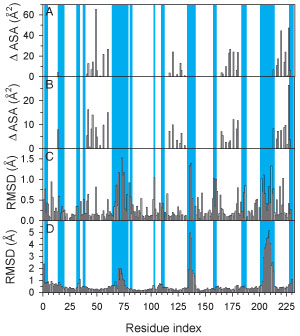
**Comparison of residue interactions with structural differences for bound 14-3-3ζ**. The average (A) and standard deviations (B) were calculated over the five 14-3-3ζ-peptide interaction profiles. These are shown aligned with the side chain RMSF (C) and the backbone RMSF (D) calculated from the five structures of bound 14-3-3ζ. Regions of residues that are highly exposed to solvent in all complex structures are indicated by the blue-shaded regions.

To further analyze the conformational changes in 14-3-3 upon binding to its multiple partners, we show the 4-panel induced-fit profile described above (Figure 8). Contrary to the results seen for the p53 DBD, 14-3-3 is much more static in its multiple interactions. All regions displaying large conformational differences across bound complexes are also highly exposed to solvent and play no direct role in mediating binding to any peptide. The plots do show several small scale structural differences – side chain rearrangements – associated with variable participation in peptide binding, particularly in the regions 40-60 and 215-230.

### 14-3-3 binding to two different partners

To gain further insight into 14-3-3 binding to different partners, we compared a pair of 14-3-3 binding peptides in detail. These two peptides, m1 and m2, were derived from two motifs, identified through the screening of peptide libraries for sequences that bound to all 14-3-3 isoforms [62]. These two peptide structures have been compared previously [61], but here we reanalyze these structural data from the order-disorder point of view.

**Figure 9:**
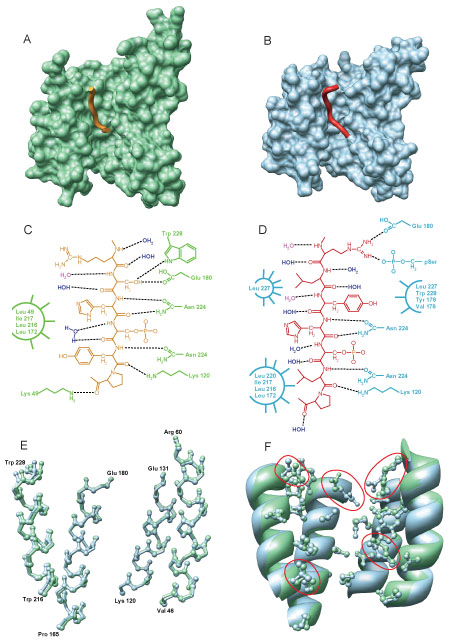
**Detailed analysis of 14-3-3ζ peptide binding**. The m1 peptide (A, orange ribbon) and m2 peptide (B, red ribbon) bound to 14-3-3 (A and B, shown by the green and blue surface, respectively). Details of 14-3-3 peptide binding are shown by a chemical schematic for the m1 peptide (C) and the m2 peptide (D), where both crystallographic waters (blue) and implicit waters (red) are shown. (E) Superposition of the backbone atoms from the 4 helices with the primary peptide binding residues for m1 (green) and m2 (blue) bound 14-3-3. (F) Superposition of ribbons of the same 4 helices showing the side chains of the residues that participate in m1 (green) and/or m2 (blue) binding.

As noted previously [61], the backbone traces of the two peptides are noticeably different even though the m1 and m2 peptides bind to essentially the same region of 14-3-3ζ (Figure 9A and B, respectively). Examining the side chain interactions of these peptides with specific 14-3-3 residues (Figure 9C and D) shows that there is difference in the location and identity of the residues involved, which is consistent with the aggregate findings (Figures 7 and 8). Similarly, distinctive hydrogen bonding patterns are exhibited between the two peptides and 14-3-3ζ and between the two peptides and bound water (Figure 9C and D). Since a cardinal feature of a structured protein is internal satisfaction of hydrogen bond donors and acceptors, these data are both consistent with the peptides being from unstructured regions of protein before binding.

The above data on the complexes suggest that 14-3-3ζ has distinct conformations when bound to the two different peptides. Overlaying the backbone structures of the four binding helices from both complexes – based on a pair-wise alignment of the complete domains – shows only minor variability in conformation, with the most occurring at the helix spanning residues 216 to 228 (Figure 9E). Finally, comparison of side chain conformation in the two complexes shows significant differences in several of 14-3-3ζ side chains (Figure 7F, residues outlined in red show significant movement) and several other minor differences. Overall, these data suggest that a difference in the conformations of some side chains with rather less difference in backbone conformations is sufficient to accommodate the binding of two different phosphopeptides by the 14-3-3ζ molecule.

## Discussion

### Use of disordered regions for binding

The large majority of the binding sites on the p53 sequence map to the disordered regions of this protein (Figure 1), indicating that intrinsic disorder commonly provides the binding sites for the various partners that associate with p53. Recent bioinformatics investigations suggest that the majority use of disorder for binding to multiple partners is quite likely to be a general result [37-41].

The p53 binding sites are often indicated on the order-disorder predictions as dips, in other words as short segments with structure tendency flanked by regions of disorder tendency on both sides. Starting from this observation, we previously developed a predictor of such regions, which we called molecular recognition features, or MoRFs, because such regions “morph” from disorder to order upon binding [87,88]. Others have used the PONDR VL-XT order/disorder plots or MoRF predictors to identify potential binding sites that were subsequently verified by laboratory experiments [89,129]. Indeed, for some of these predicted examples, the regions did indeed form helix upon binding to their partners [130,131]. By greatly enlarging the training set, we recently improved the MoRF predictor. Interestingly, when tested against several order-disorder predictors including ones from other laboratories, PONDR VL-XT, gave the clearest indication of binding sites within disordered regions [90].

Others developed a sequence-based approach to identify short, conserved recognition sites, called eukaryotic linear motifs (ELMs) [132,132-134]. While MoRFs are identified by general order/disorder tendencies and while ELMs are identified by motif discovery from sequence analysis, the resulting binding sites identified by both methods share several features [135]. The use of different residues in the same disordered fragment for one-to-many signaling leads to a potential problem with the ELM model. That is, the concept behind ELMs is that each ELM uses a common set of amino acids for binding to different partners. These common amino acids therefore show up as an over-represented pattern leading to a “linear motif”. What if a region used to bind to multiple partners uses different secondary structures and different amino acids? In such a case, the residues in the “linear motif” would not necessarily be over-represented. It will be interesting over time to determine whether ELMs having stronger signals use a reduced set of structures for their interactions.

While the observed binding sites in the disordered regions of p53 have a localized tendency for ordered structure, not all disorder-associated binding sites exhibit such features. We have found many binding sites that are associated with high disorder prediction values across the entire spans of the binding sites, one example of which was recently published [136]. Many of these dipless MoRFs form irregular structures upon binding with their partners, and often such binding regions are rich in proline. Our recent study of the complexes that form when various disordered segments bind to ordered partners indicates that the disorder-associated binding regions have distinct sequence features, even when the bound structure is irregular or sheet instead of helix, and so it should be feasible to develop a specific predictor for each of the different types of MoRFs [137].

### One-to-many signaling

Date hubs bind to different proteins at different times. Figure 3 shows how a single region of p53 binds to four different partners. The amino acids involved in each interaction show a significant overlap and no two of these interactions could exist simultaneously. Furthermore, the same residues adopt helix, sheet, and two different irregular structures when associated with the different partners. Finally, the same amino acids are buried to very different extends in each of the molecular associations. These results show very clearly how one segment of disordered protein can bind to multiple partners via the ability to adopt distinct conformations.

The idea that one segment of protein can adopt different secondary structures depending on the context is not new. Many unrelated proteins have identical subsequences of length six, and sometimes even up to length eight, with the same sequences often adopting different secondary structures in different contexts [138,138-140]. Such sequences have been called chameleons for their ability to adopt different structures in different environments [139-145]. Chameleon behavior could be an important feature that enables one disordered region to bind to multiple partners. With different secondary structures and with different side chain participation in the different complexes, it is as if one sequence can be “read” in multiple ways by the various binding partners.

Chameleon behavior occurs for short peptides (octamers), for longer protein fragments and even for entire proteins. For example, the 17 residues-long arginine-rich RNA binding domain (residues 65–81) of the Jembrana disease virus (JDV) Tat protein recognizes two different transactivating response element (TAR) RNA sites, from human and bovine immunodeficiency viruses (HIV and BIV, respectively). The JDV segment adopts different conformations in the two RNA contexts and uses different amino acids for recognition [142]. In addition to the above conformational differences, the JDV domain requires the cyclin T1 protein for high-affinity binding to HIV TAR, but not to BIV TAR [142]. Another protein with chameleon properties is human α-synuclein, which is implicated in Parkinson's disease and in a number of other neurodegenerative disorders known as synucleinopathies. This protein may remain substantially unfolded, or it may adopt an amyloidogenic partially folded structure, or it may fold into α-helical or β-strand species, including both monomeric and oligomeric species. In addition, this protein can form several morphologically different types of aggregates, including oligomers (spheres or doughnuts), amorphous aggregates, and amyloid-like fibrils [34].

Such chameleon sequences likely underlie the multiple specificity binding sites common in p53. For a quick calculation of the implied degree of interface overlap, assume that each residue in a region has equal probability to interact with a partner and consider the C-terminus of p53. The disordered C-terminus (~100 residues) associates with at least 44 distinct partners. The average length of a binding site in this region is ~14 residues, which means that on average only 100/14=7 partners bind at any given residue in the C-terminus. This simple back-of-the-envelope calculation suggests that multiple specificity sequences may be the rule for p53 interactions, rather than a curiosity of a single region. However, available data suggests that interactions do not overlap in a random fashion, but rather interactions are localized to specific regions. For example, consider that the majority of the structures available for the C-terminus of p53 involved the same region of sequence. Therefore, the back-of-the-envelope calculation provides an approximate minimum degree of overlap, where the actual degree of overlap is likely much higher. This idea, which is an extension of a previous proposal [117], further suggests a general mechanism by which hub proteins could bind to such a large multitude of partners, which cannot be explained from the view point of interaction between two structured proteins [51].

Finally, the p53 DBD offers a counter example to the disorder-based view of date hubs. That is, it uses the same or similar face of its globular structure to bind to multiple partners. While the p53 DBD is a folded protein, it does exhibit some remarkable structural differences when bound to difference partners. It seems unlikely that these *local* regions of the p53 DBD structure are well folded in isolation, otherwise the association rate of some or all of these complexes would be relatively low. This idea is supported by the finding that the p53 DBD is only marginally stable at physiological temperature [146]. Therefore, it is plausible that these regions of the monomeric DBD are only transiently folded in solution, where crystallization conditions cause a shift toward the folded state in monomeric crystal structures. The double NIA-NMA plot data (Figure 2B) does not contradict this idea, since it is limited to global analysis and this idea only applies to local regions of the DBD. This idea is conjecture and further experimental or simulation evidence is needed to test this idea. In any event, however, the p53 DBD demonstrates that even proteins generally thought to be well folded, structural changes can still occur in association with multiple specificity.

### Many-to-one signaling

In 14-3-3, a common binding groove in a structured dimeric protein can be fitted by multiple, distinct sequences provided by many different binding partners. A recent bioinformatics study [63] found that14-3-3 proteins, and the 14-3-3 binding regions in particular, are predicted to be highly disordered by multiple disorder prediction methods. The authors proposed that 14-3-3 recognition generally involved coupled binding and folding of the recognition region. Our results support this conclusion because the backbone of m1 and m2 peptides are highly hydrated in the bound state (Figure 9C and D), indicating that the binding peptide is likely to be unstructured prior to binding [83].

One idea is that 14-3-3 holds its bound partner in a non-active state [63]. Even though 14-3-3 likely binds to disordered regions in its partners (data herein and [63]), this idea of blocking the active structure could still be true. For example, the productive state of 14-3-3's partner might involve the binding of the partner to a second partner via the same disordered region that binds to 14-3-3, in which case 14-3-3 binding would prevent the formation of the productive complex. Another possibility is that the disordered region exhibits an equilibrium between a bound state that activates the protein and an unbound state that inactivates the protein. The association of the unbound disordered region with 14-3-3 would then hold 14-3-3's partner in the non-productive state as proposed previously.

We previously suggested that disordered segments with different sequences could use their flexibility to bind to a common binding site, thereby facilitating many-to-one signaling [35]. The multiple recognition of 14-3-3 depends on this mechanism to a considerable degree, with the different peptides taking different paths through the binding cleft and interacting with binding site residues in distinct ways (Figure 6B).

In addition, structured proteins also have a degree of flexibility, and so the binding site backbone and side chain residues can undergo shifts (induced-fit mechanisms) to help accommodate interactions with distinct sequences (Figure 6 and 8). Thus, induced-fit mechanisms are important for structured protein interactions with different partners whether the partners are structured or intrinsically disordered.

The induced-fit mechanisms observed for 14-3-3 and the DNA binding domain of p53 are commonly observed in other situations. For example, tethering, in which a peptide is covalently linked to its protein target to allow detection of low affinity interactions, often results large-scale side chain movements concomitant with peptide binding [147]. Also, when many different MoRFs and their binding partners are examined, induced-fit movements in the structured partners are very commonly observed [137]. Similarly, small backbone shifts and side chain conformational changes are both important for 14-3-3's ability to bind multiple partners. For all of these examples, the associations involve coupled binding and folding for the disordered peptide partner coupled with a near universal classical induced fit for the structured side of the partnership.

### One-to-many signaling vs. many-to-one signaling

The p53 C-terminus and 14-3-3 use intrinsic disorder differently with regard to enabling multiple binding specificities. In p53, drastic conformational changes enable distinct surfaces to be exposed to binding partners. In 14-3-3, subtle differences in 14-3-3 conformation and peptide binding locations enable multiple specificities. Why would nature use one mechanism rather than the other for a particular biological role? The interactions of p53 serve to activate or inhibit its primary role as a transcription regulator, while 14-3-3 alters the functions or subcellular localization of many proteins. From this, one can make some highly speculative proposals: (1) disorder binding regions play a passive role in regulation by providing a specific binding site – i.e. the disordered regions are the identification sites of the protein to be regulated [148] – and (2) ordered proteins play the active role – i.e. altering the activity of the proteins they bind to – where recognition of disordered regions allows for a generalized specificity so that a single protein can alter the activity of many others. Validation of the accuracy and generality of these ideas requires further study.

## Conclusions

Here we have examined the mechanisms of multiple specificities in two date hub-like hub proteins. Evidence here and elsewhere [37-41], suggests that disordered regions may be an extremely common mechanism by which hub proteins bind to their multitude of partners. The specific examples of p53 and 14-3-3 contrasts the mechanisms by which disorder facilitates multiple recognition, where the former involves drastic conformational differences in a single disordered region and the later involves a variety of subtler changes in order to recognize multiple disordered regions. Finally, it is proposed that the differences between the binding of the disordered region of p53 and the binding of disordered regions to 14-3-3 may have implications for the biological roles of both types of interactions.

## Methods

### PONDRs VL-XT and VSL1

Predictions of intrinsic disorder in HPV proteins were performed using a set of PONDR^®^ (Predictor Of Natural Disordered Regions) predictors, VL-XT and VSL2. PONDR® VL-XT integrates three feed forward neural networks: the Variously characterized Long, version 1 (VL1) predictor from Romero *et al*. 2001 [84], which predicts non-terminal residues, and the X-ray characterized N- and C- terminal predictors (XT) from Li *et al.* 1999 [149], which predicts terminal residues. Output for the VL1 predictor starts and ends 11 amino acids from the termini. The XT predictors output provides predictions up to 14 amino acids from their respective ends. A simple average is taken for the overlapping predictions; and a sliding window of 9 amino acids is used to smooth the prediction values along the length of the sequence. Unsmoothed prediction values from the XT predictors are used for the first and last 4 sequence positions.

The recently developed Various Short-Long, version 1 (PONDR®-VSL1) algorithm is an ensemble of logistic regression models that predict per-residue order-disorder [85,150]. Two models predict either long (>30 residues) or short (<15 residues) disordered regions based on features similar to those used by VL-XT. The algorithm calculates a weighted average of these predictions, where the weights are determined by a meta-predictor that approximates the likelihood of a long disordered region within its 61-residue window. Predictor inputs include PSI-blast profiles [151], and PHD [152], and PSI-pred [153] secondary structure predictions.

### Structure surface and complex interface analysis

Solvent accessible surface area (ASA) was calculated from atomic protein structure numerically using the double cubic lattice method [154] as implemented in the Biochemical Algorithms Library [155]. Using this algorithm, ASA of residues and entire chains can be calculated.

To determine interface areas, for example between two chains, the ASA of each individual chain is calculated, as well as the ASA of the complex. The interface area is then calculated as the change in ASA (ΔASA), i.e. the sum of the individual chain ASA minus the complex ASA. Residues directly involved in interactions were identified from molecular structures as residues with a ΔASA greater than 1 Å^2 ^[112,113]. All calculations used a probe radius of 1.4 Å, which roughly corresponds to the size of a water molecule.

### Order-disorder evaluation from known structure

The work of Gunasekaran et al. has previously shown that, in many cases, the order-disorder state of a protein prior to complex formation is reflected in the complex structure [117]. Specifically, a plot of the normalized monomer area (NMA) – ASA divided by the number of monomer residues – versus the normalized interface area (NIA) – ΔASA divided by the number of monomer residues – effectively distinguishes between ordered and disordered monomers using a linear boundary. This effectiveness of this NMA-NIA plot has been validated on an expanded dataset and an optimal linear boundary has been estimated and evaluated (Oldfield et al., manuscript in preparation). The equation for the novel boundary is:

<NMA>=157.43-3.51<NIA>

Since the NMA-NIA plot can only represent one partner of a complex, the double NMA-NIA plot was developed to simultaneously represent both monomers of a binary complex – or complexes that can be treated as binary, such as two monomers bound to a dimer. Rather than plotting the NMA and NIA directly, the Euclidean distance to the order-disorder boundary is calculated, where disordered monomers have a positive distance and ordered monomers have a negative distance. Then the boundary distances of each monomer in a binary complex can be plotted against each other to give an overall order-disorder prediction for the complex. The double NMA-NIA plot is covered in more detail elsewhere (Oldfield et al., manuscript in preparation).

### Other structure calculations

The root mean squared fluctuation (RMSF) is a commonly used measure of variability across multiple structure alignments. Here, RMSF of the protein backbone is approximated as the RMSF of the C_α_ atoms. The equation used is

$$RMSF_{i}=\sqrt{\frac{1}{N}{\sum_{j=1}^{N}\left(C_{\alpha_{j,i}}-\bar{C_{\alpha_{i}}}\right)}^{2}}$$

where $C_{\alpha_{j,i}}$ is the position vector of the i^th^ C_α_ atom of the j^th^ complex and $\bar{C_{\alpha_{i}}}$ is the averaged position for the i^th^ amino acid from the multiple sequence alignment of N structures. The program MultiProt [156] was used to generate the multiple sequence alignments for RMSF calculation and structure rendering.

To estimate side chain conformation variability among multiple protein structures, the RMSF of side chain residues was calculated. In this calculation, the residue atoms C_α_,C_β_, backbone carbonyl carbon, and backbone nitrogen were used to align a residue to a selected reference residue of the same type. Thus aligned, the RMSF was calculated over side chain carbons beyond the C_β_. Consequently, no side chain RMSF was calculated for Glycine or Alanine residues. The RMSF was also corrected for the number of atoms in the side chain beyond the C_β_.

The solvent accessibility of individual residues was calculated relative to an extended Gly-X-Gly model peptide [157], which gives a conservative estimate of relative solvent exposure, i.e. underestimates relative solvent exposure. Residues exposed to solvent were defined as those with an accessible surface area at least 40% of that of the reference area for that residue type. This cutoff is arbitrary, but cutoffs for solvent exposed residues as low as 20% have been used by others, e.g. [158]. Solvent exposures were calculated in the context of binary complexes, which is valid for p53 complexes. In 14-3-3 complexes, 14-3-3 forms homotypic dimmers in addition to binding to phosphopeptides, so residues found to be highly solvent exposed are either actually exposed to solvent or involved in the homodimer interface.

## Competing interests

The authors declare that they have no competing interests.

## Authors' contributions

CJO has done the computational analysis, designed figures and contributed to the manuscript writing. JM, MQY and JYY were involved in finding and analyzing p53 and 14-3-3 binding partners. VNU was involved in planning of experiments, contributed to the manuscript writing and revised the final version. AKD was involved in design and planning of all the experiments, drafted the manuscript and headed the project. All authors have read and approved the final manuscript.
